# Supplementary material for: Machine Learning‐Driven Nanopore Sensing for Quantitative, Label‐Free miRNA Detection
Source: Small Methods. 2026 Jan 19;10(4):e02335. doi: 10.1002/smtd.202502335 (PMC12929938; doi:10.1002/smtd.202502335)
Supplement: Supplementary file 1 — Supporting File: smtd70480‐sup‐0001‐SuppMat.pdf. [file SMTD-10-e02335-s001.pdf]

Supporting Information for

Machine Learning-Driven Nanopore Sensing for Quantitative, Label-Free miRNA Detection

Caroline Koch[1,2‡], Seshagiri Sakthimani[1‡], Victoria Maria Noakes[1], Miruna Cretu[1], David Newman[3], Richard Gutierrez[3], Mark Bruce[3], Julia Gorelik[4], Nadia Guerra[2], Joshua B. Edel[1], Aleksandar P. Ivanov[1]**

1 Department of Chemistry, Molecular Science Research Hub, Imperial College London, White City Campus, 82 Wood Lane, London, W12 0BZ, UK

2 Department of Life Science, Sir Alexander Fleming Building, Imperial College London, South Kensington Campus, Imperial College Rd, London, SW7 2AZ, UK

3 Oxford Nanopore Technologies, Oxford Science Park, Edmund Halley Road, Oxford OX4 4DQ, UK

4 National Heart and Lung Institute, ICTEM, Imperial College London, Hammersmith Campus, 72 Du Cane Rd, London, W12 0NN, UK

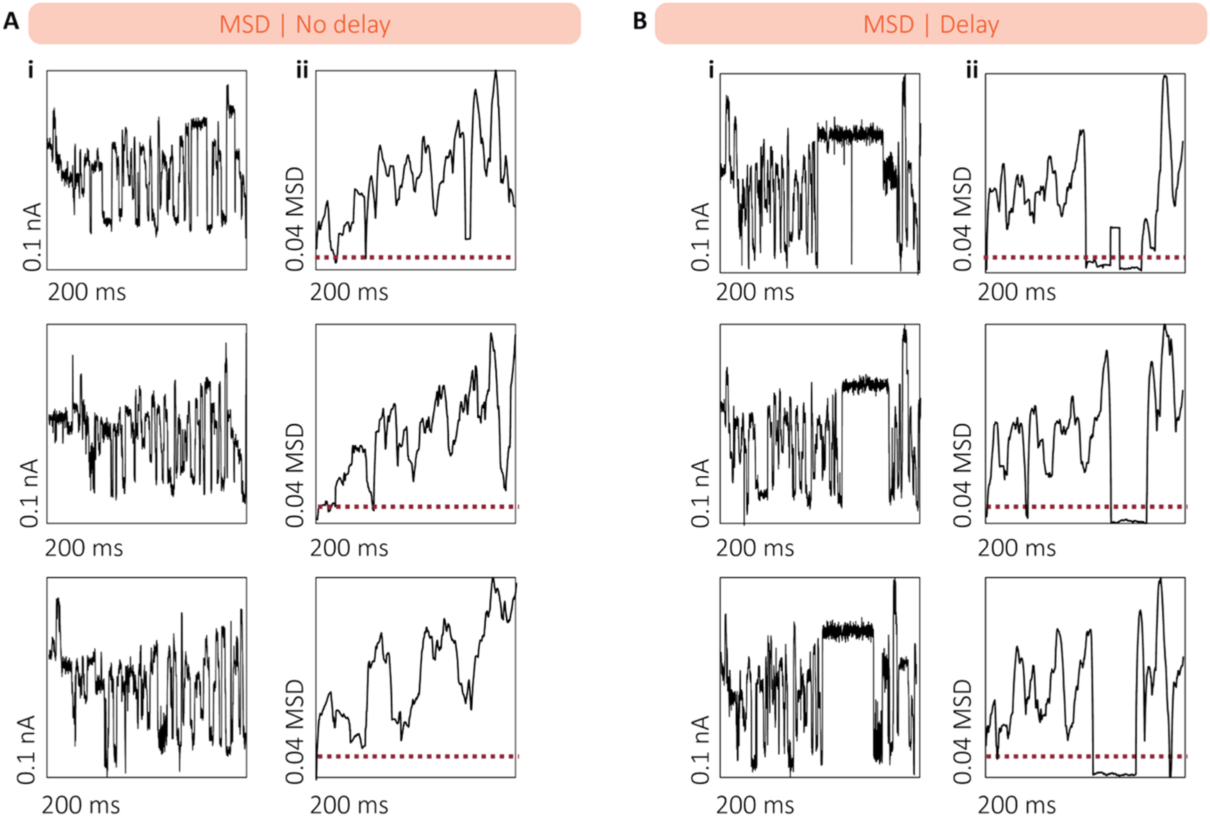

Supplementary Data 1 | Examples of delayed and non-delayed events using the MSD method. (A) Representative non-delayed events showing the **(i)** raw current signal and **(ii)** the MSD signal. **(B)** Representative delayed events showing the **(i)** raw current signal and **(ii)** the MSD signal.

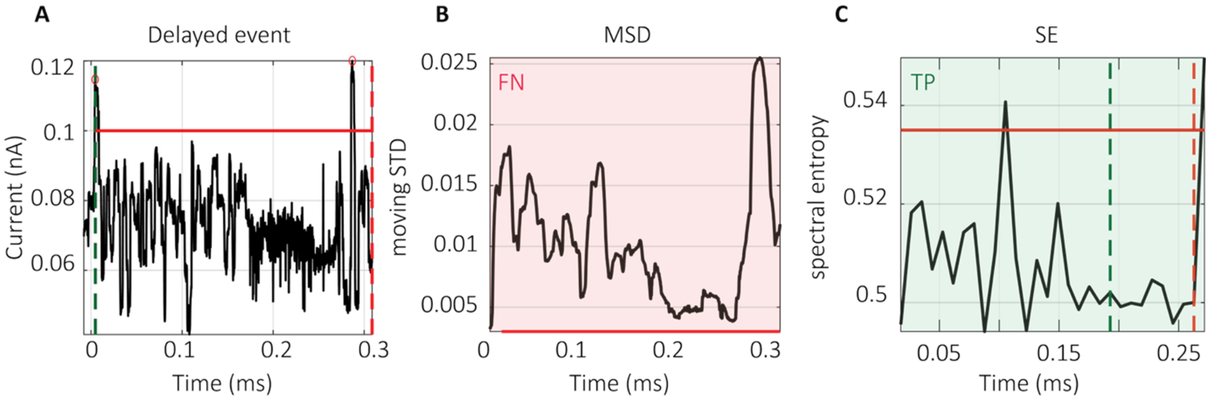

Supplementary Data 2 | Examples of failed delay detection. (A) Delayed events exhibiting noisy delay signatures**. (B)** MSD fails to detect the delay due to elevated signal variability. **(C)** SE correctly classifies the same event as delayed, demonstrating improved robustness to noise.

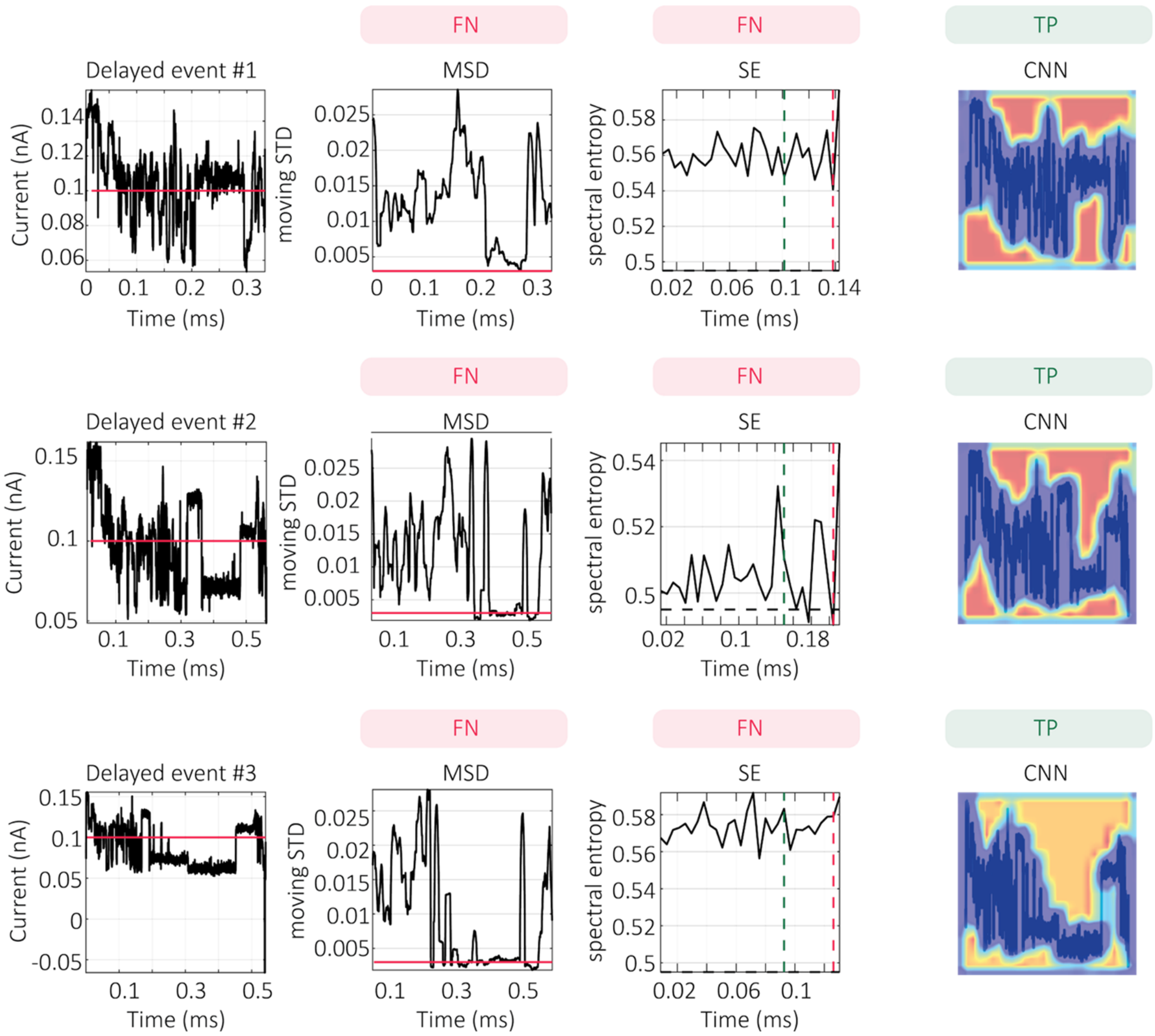

Supplementary Data 3 | Comparison of threshold-based feature extraction methods (MSD/SE) and CNN-based classification for noisy delayed events. Representative delayed nanopore translocation events (left column) are shown as raw ionic current traces. Each event is analysed using three approaches: (i) MSD (middle-left column), (ii) SE (middle-right column), and (iii) a CNN model (right column). The CNN operates directly on the raw current traces, with corresponding activation heatmaps highlighting regions that contribute most strongly to the classification decision. In contrast to MSD and SE, which depend on manually defined thresholds and windowing parameters and therefore misclassify several delayed events as false negatives (FN), the CNN learns hierarchical, noise-robust features directly from the signal. This enables reliable identification of delayed events across diverse signal morphologies, including noisy delay regions (event #1), step-wise delays (event #2), and events with large current amplitudes (event #3), resulting in correct true-positive (TP) classification.

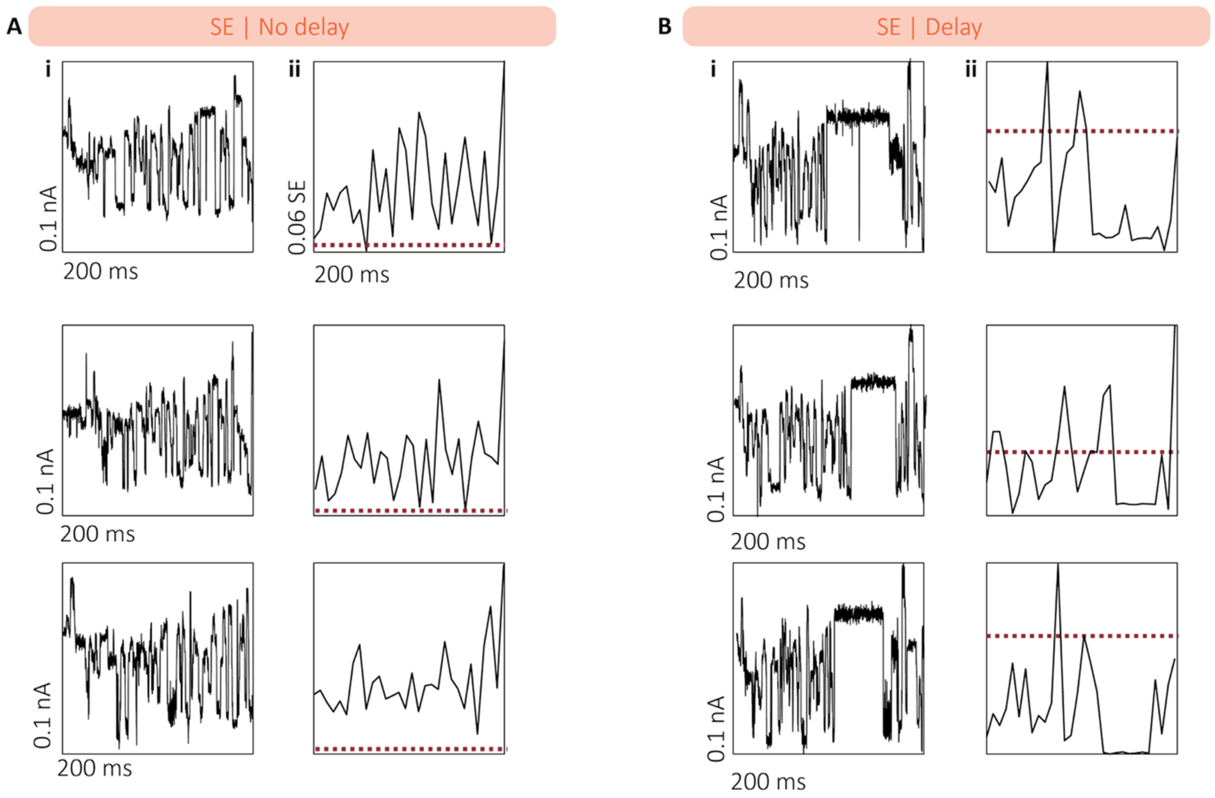

Supplementary Data 4 | Examples of delayed and non-delayed events using the SE method. **(A)** Representative non-delayed events showing the **(i)** raw current signal and **(ii)** the MSD signal. **(B)** Representative delayed events showing the **(i)** raw current signal and **(ii)** the MSD signal.

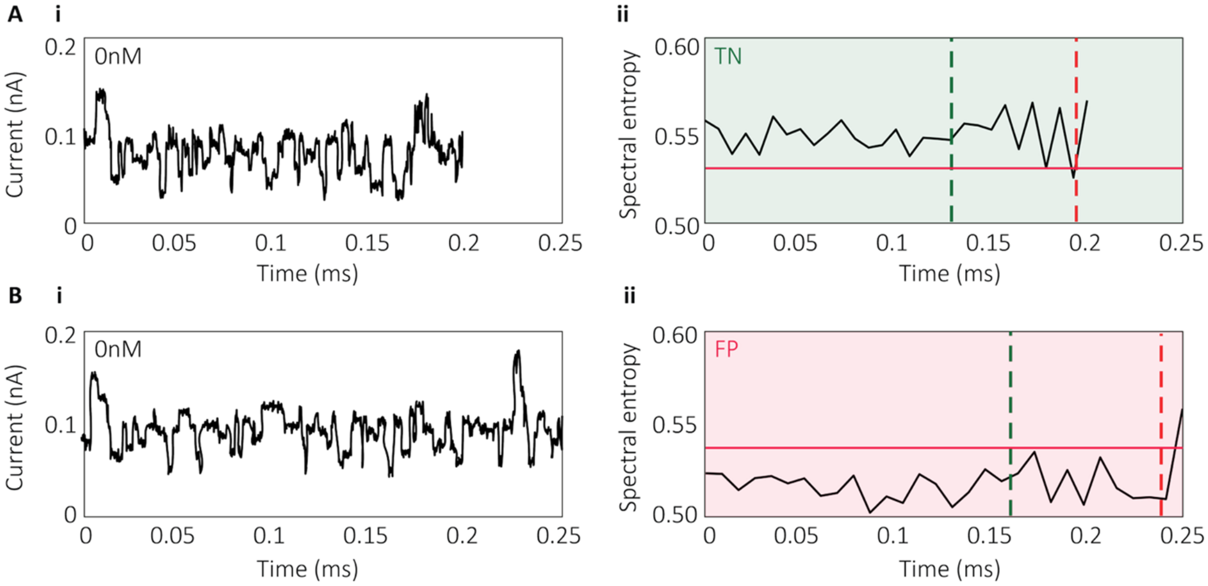

Supplementary Data 5 | Misclassification of delayed events using SE. (A) True negative (TN): a non-delayed event correctly classified by SE. **(B)** False positive (FP): a non-delayed event misclassified as delayed due to increased dwell time, highlighting a limitation of SE in distinguishing signal features.

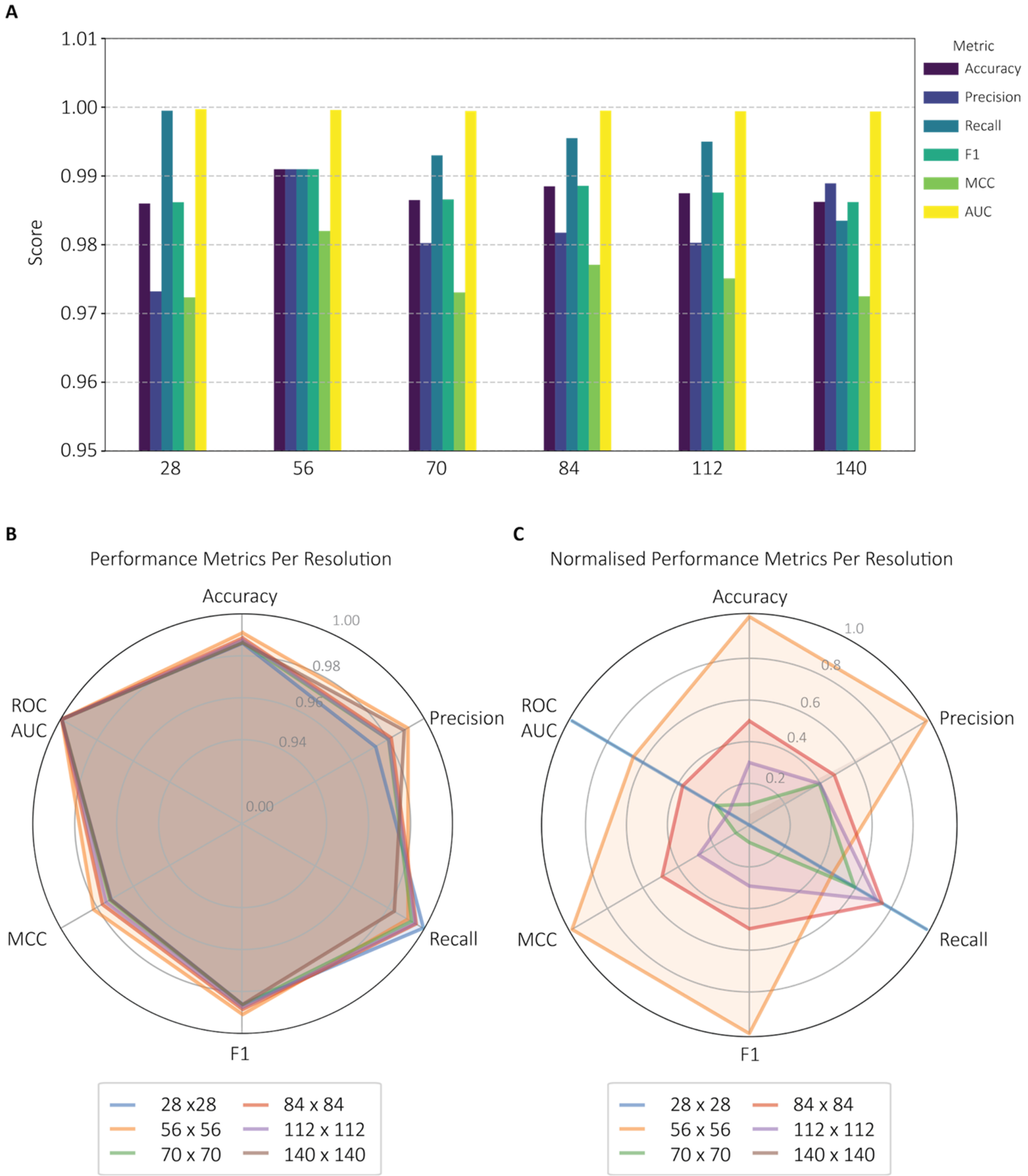

Supplementary Data 6 | CNN performance across varying image resolutions. (A) Bar plot comparing raw performance metrics for CNN models trained at six image resolutions 28 × 28, 56 × 56, 70 × 70, 84 × 84, 112 × 112, and 140 × 140 pixels) **(B)** Radar plots of unscaled metric values per resolution. **(C)** Radar plots of normalised metrics (min–max scaling) to highlight relative performance across resolutions.

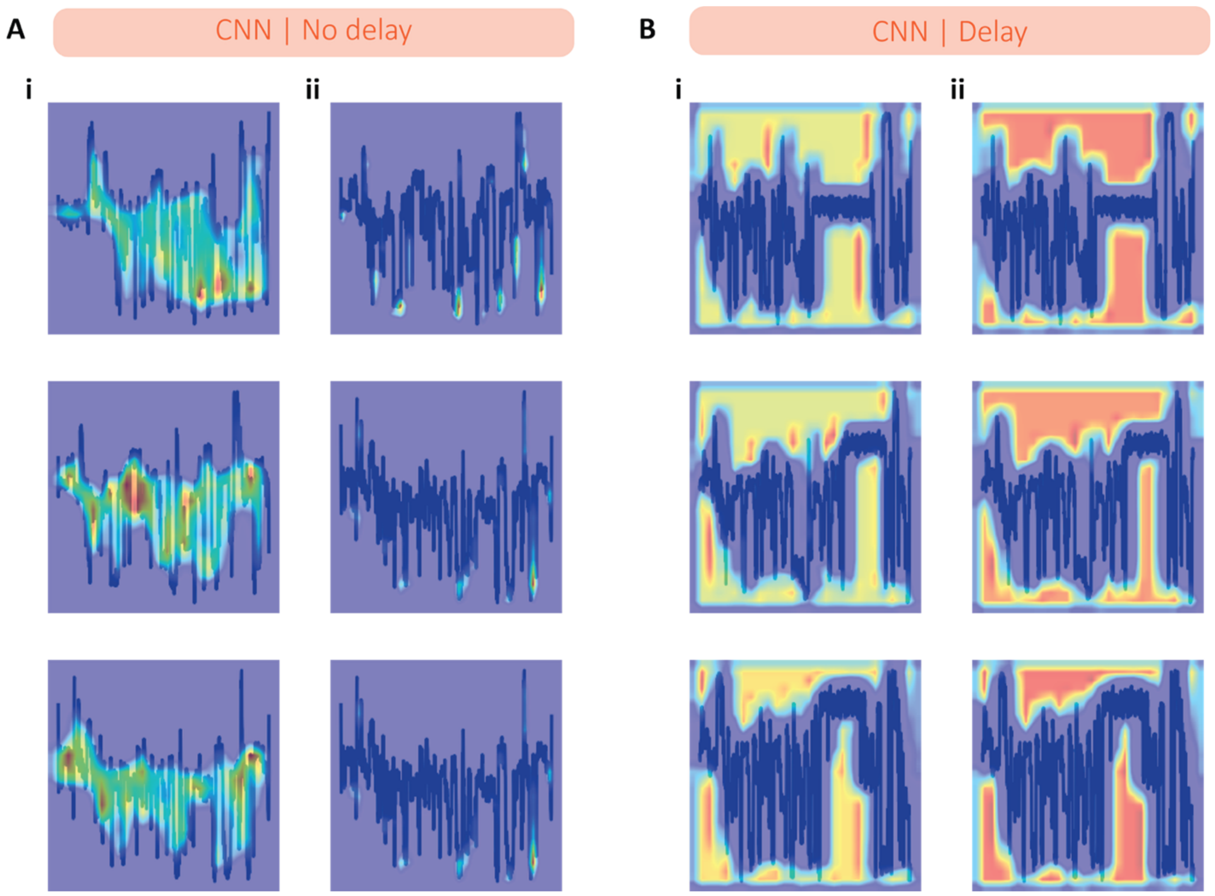

Supplementary Data 7 | Grad-CAM versus Grad-CAM++ visualisations. (A) Non-delayed and **(B)** delayed event comparison of saliency maps generated using **(i)** Grad-CAM and **(ii)** Grad-CAM++ for non-delayed events. Grad-CAM was selected for its ability to consistently highlight the full signal envelope, resulting in clearer, more coherent visualisations across both delayed and non-delayed conditions.

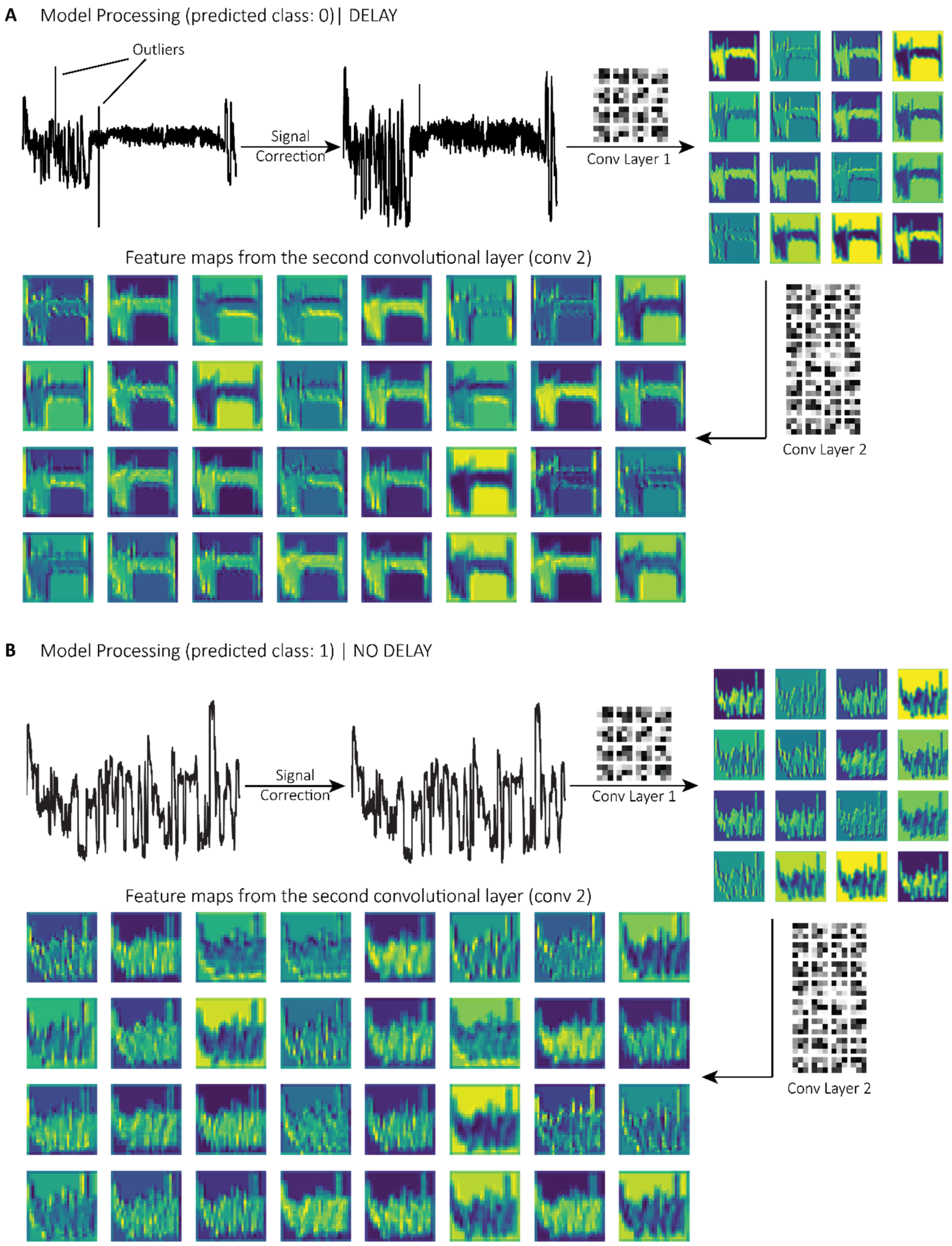

Supplementary Data 8 | CNN signal processing workflow. Illustration of the CNN pipeline, showing image transformation through successive convolutional layers. **(A)** Example of a delayed event containing outliers, processed through the signal correction algorithm. **(B)** Example of a clean non-delayed event. The image structure becomes abstracted after the second convolutional layer and is therefore not shown.

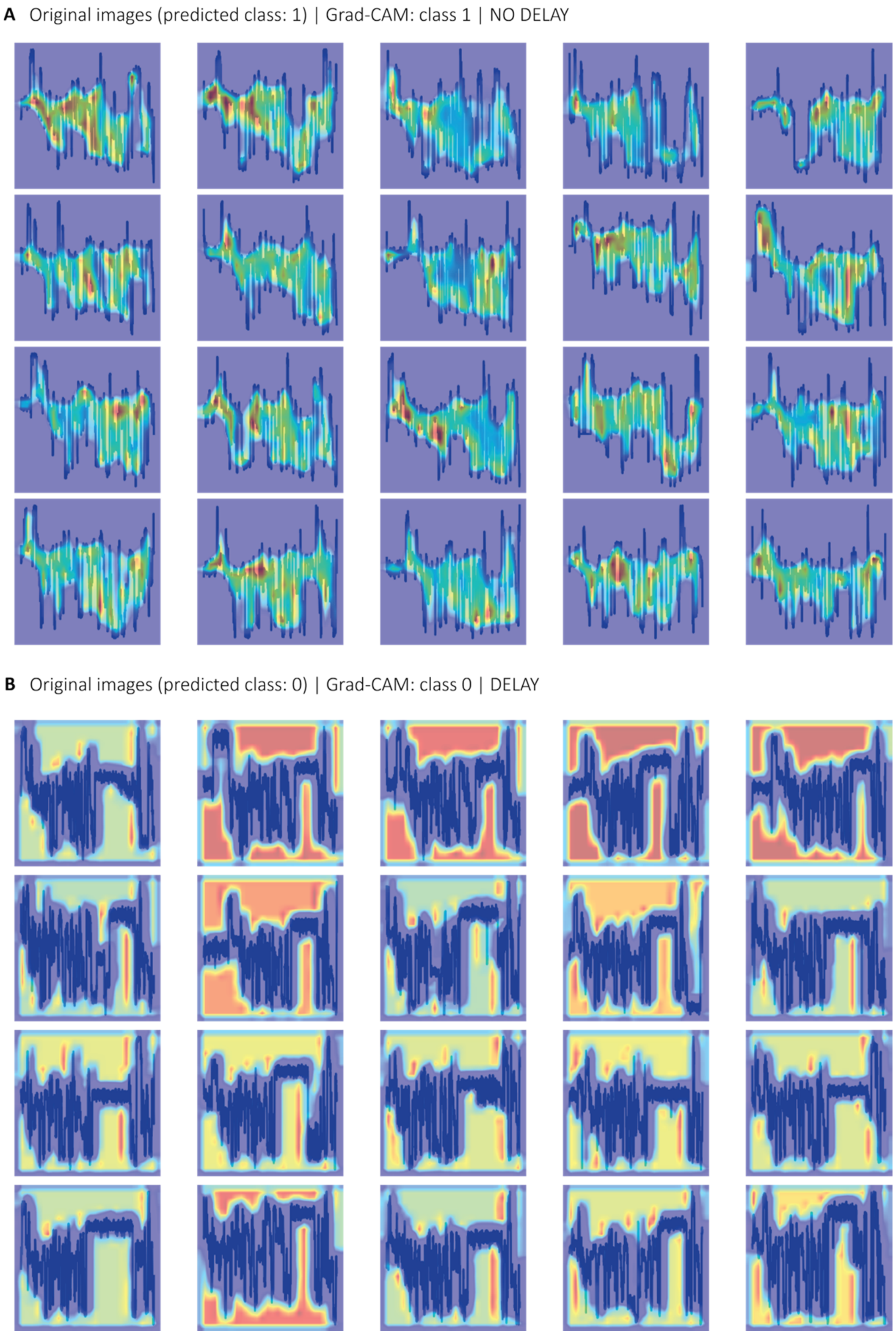

Supplementary Data 9 | Grad-CAM analysis of CNN attention across multiple events (A) Saliency maps for 20 non-delayed events. **(B)** Saliency maps for 20 delayed events. The CNN consistently focuses on signal regions associated with delay, confirming the model's interpretability and robustness across diverse examples.

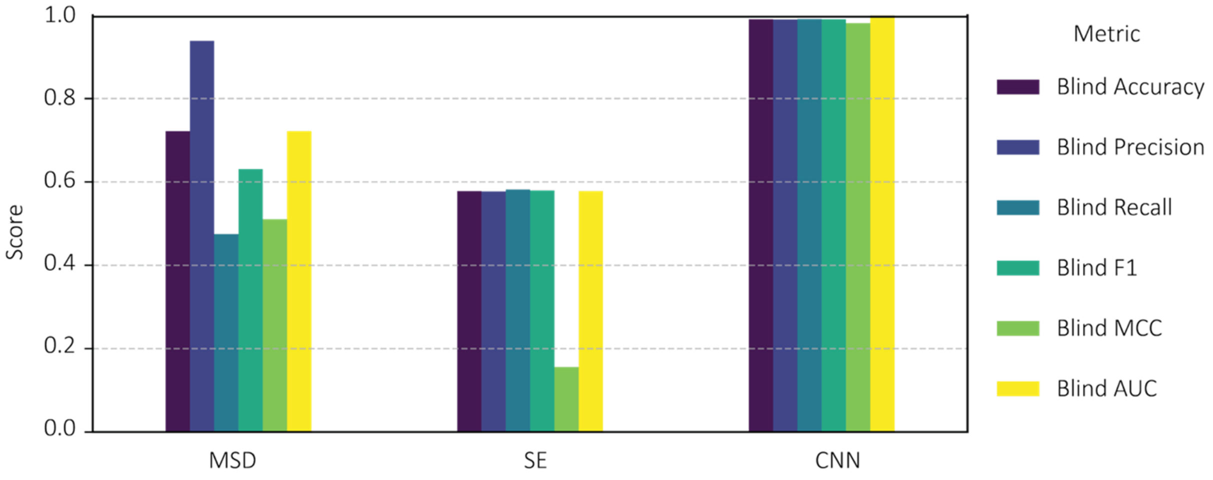

Supplementary Data 10 | Bar plot of key performance metrics. All three approaches (MSD, SE, and CNN) were compared for their accuracy, precision, recall, F1 score, MCC, and ROC AUC, with CNN showing the best performance across these metrics.

Supplementary Data 11 | DNA-barcoded probe sequences and miRNA targets used for CNN training and testing. Table listing all DNA-barcoded probes and their corresponding miRNA targets. Probes and targets highlighted in blue and marked with an asterisk (*) were included in the training dataset.

DNA-barcoded probe sequence	miRNA target	miRNA sequence
/5Phos/CCT AGT TCC GCT GGG ATC GCT ACG CCT TCG GCT CGT AAT CAT AGT CGA GT/iSpC3//iSpC3/G TTC ACC AAT CAG CTA AGC TCT	hsa-miR-27b-5p*	rArGrA rGrCrU rUrArG rCrUrG rArUrU rGrGrU rGrArA rC
/5Phos/CCT AGT TCC GCT GGG CTA GTG CGC AGT TGT CTC GGC GGA GTT GAG ACT GA/iSpC3//iSpC3/A CCC ACC GAC AGC AAT GAA TGT T	hsa-miR-181b-5p*	rUrArG rCrUrU rArUrC rArGrA rCrUrG rArUrG rUrUrG rA
/5Phos/CCT AGT TCC GCT GGG GTT CAC ATC AAG GTC ATA CCG CGA GTT CTA TTT TA/iSpC3//iSpC3/C AGT GTG CGG TGG GCA GGG GCT	hsa-miR-210-5p*	rArCrC rUrGrG rCrArU rArCrA rArUrG rUrArG rArUrU rU
/5Phos/CCT AGT TCC GCT GGG GCT TGG GGG ATA GAT GTG CCC CGC GCA TCG GAC CT/iSpC3//iSpC3/C GCG TAC CAA AAG TAA TAA TG	hsa-miR-126a-5p*	rUrGrU rArArA rCrArU rCrCrC rCrGrA rCrUrG rGrArA rG
/5Phos/CCT AGT TCC GCT GGG AAC CTT AGG GGC CTC GAA TCT TTG AGA CGA CTA GG/iSpC3//iSpC3/C TAT CTG CAC TAG ATG CAC CTT A	hsa-miR-18b-5p*	rArGrC rCrCrC rUrGrC rCrCrA rCrCrG rCrArC rArCrU rG
/5Phos/CCT AGT TCC GCT GGG ATG ACA CAC GTT TTC GAT AGG GAC GCC GAC TTT AA/iSpC3//iSpC3/T GGA CGT TTG CAG GGG AGG TGG	hsa-miR-1306-5p	rUrGrU rArArA rCrArU rCrCrU rArCrA rCrUrC rUrCrA rGrC
/5Phos/CCT AGT TCC GCT GGG TGA TAA TAA GAC CTG ACA GAC AAT AGG GAG AAC TC/iSpC3//iSpC3/C GCG TAC CAA AAG TAA TAA TG	hsa-miR-126-5p	rArArC rArUrU rCrArU rUrGrC rUrGrU rCrGrG rUrGrG rGrU
/5Phos/CCT AGT TCC GCT GGG ATT AGC GGA ACC AAA CCC AGG AAG GCT TGA AGG CG/iSpC3//iSpC3/A TAC ATA CTT CTT TAC ATT CCA	hsa-miR-1-3p	rUrArG rCrArC rCrArU rCrUrG rArArA rUrCrG rGrUrU rA
/5Phos/CCT AGT TCC GCT GGG TAA TTA CTG CCC CAC CAT GAC ATT TTA ATA GCA GT/iSpC3//iSpC3/C TAA CTG CAC TAG ATG CAC CTT A	hsa-miR-301a-5p	rCrArU rUrArU rUrArC rUrUrU rUrGrG rUrArC rGrCrG

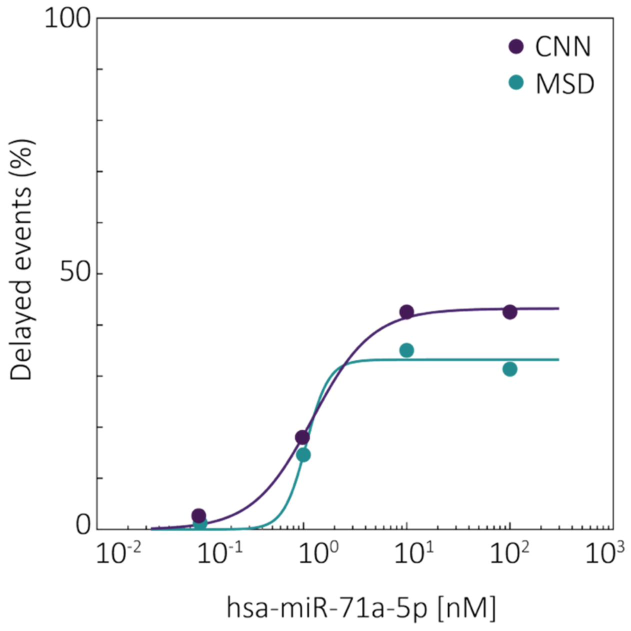

Supplementary Data 12 | Concentration-response curves for additional miRNA. The percentage of delayed events is shown as a function of hsa-miR-71a-5p concentration (0-100 nM), fitted using the Hill equation. The MSD method yielded n_h = 4.00, K_e = 1.06 nM, V_{max} = 33.20%, and the CNN model yielded n_h = 1.50, K_e = 1.22 nM, V_{max} = 43.18%.
